# Does Computer-Assisted Femur First THR Improve Musculoskeletal Loading Conditions?

**DOI:** 10.1155/2015/625317

**Published:** 2015-10-25

**Authors:** Tim A. Weber, Sebastian Dendorfer, Joachim Grifka, Gijsbertus J. Verkerke, Tobias Renkawitz

**Affiliations:** ^1^Faculty of Mechanical Engineering, Laboratory for Biomechanics, Ostbayerische Technische Hochschule Regensburg, 93053 Regensburg, Germany; ^2^Department of Orthopaedic Surgery, Regensburg University Medical Center (UKR), 93077 Bad Abbach, Germany; ^3^Department of Rehabilitation Medicine, University Medical Center Groningen (UMCG), University of Groningen, Antonius Deusinglaan 1, 9713 AV Groningen, Netherlands; ^4^Department of Biomechanical Engineering, University of Twente, Drienerlolaan 5, 7522 NB Enschede, Netherlands

## Abstract

We have developed a novel, computer-assisted operation method for minimal-invasive total hip replacement (THR) following the concept of “femur first/combined anteversion,” which incorporates various aspects of performing a functional optimization of the prosthetic stem and cup position (CAS FF). The purpose of this study is to assess whether the hip joint reaction forces and patient's gait parameters are being improved by CAS FF in relation to conventional THR (CON). We enrolled 60 patients (28 CAS FF/32 CON) and invited them for gait analysis at three time points (preoperatively, postop six months, and postop 12 months). Data retrieved from gait analysis was processed using patient-specific musculoskeletal models. The target parameters were hip reaction force magnitude (hrf), symmetries, and orientation with respect to the cup. Hrf in the CAS FF group were closer to a young healthy normal. Phase-shift symmetry showed an increase in the CAS FF group. Hrf orientation in the CAS FF group was closer to optimum, though no edge or rim-loading occurred in the CON group as well. The CAS FF group showed an improved hrf orientation in an early stage and a trend to an improved long-term outcome.

## 1. Introduction

Total hip replacement (THR) is one of the most successful operations of the 20th century [1]. Instability and early aseptic loosening are the two most common early complications following THR [2–5]. Biomathematical calculations have shown that prosthetic instability can be reduced by regarding stem and cup as coupled partners in a biomechanical system [6]. In this context, several authors have proposed starting with the preparation of the femur and then transferring the orientation of the stem relative to the cup intraoperatively (“femur first,” “combined anteversion”) in order to minimize the risk of impingement and dislocation [7–10]. We have developed a novel, computer-assisted operation method for THR following the concept of “femur first/combined anteversion” (CAS FF), which incorporates various aspects of performing a functional optimization of the prosthetic stem and cup position [11–13]. Goal of this study was to compare the hip reaction forces (hrf) and their orientation, which are known to influence implant survivorship [14–16], between CAS FF and conventional THR (CON). One method to analyze hrf is to employ instrumented implants (II) [17, 18]. This method is regarded as the gold standard, since it is the only way to measure such forces in vivo; however it bears the disadvantage of being highly invasive. This limits this method to only small sample sizes, making statistical analysis and predictions challenging. Novel computational methods like musculoskeletal modeling (MM) have the potential to accurately predict hrf while being noninvasive [19]. Validation of such models has been achieved by comparing computed entities to measured ones [20]. After validation has been achieved the models can be employed to investigate larger collectives [16]. Often such studies focus on activities of daily living (ADL) such as walking [21]. By combining experimental data as retrieved from motion capture gait analysis, medical imaging, and MM it is possible to build anatomical correct models that represent the patient accurately [22], allowing the computation of muscle forces and hip reaction forces in a patient-specific manner [19]. Such data can help to further improve implant design and can be used for measuring the outcome after THR [21]. Analyzing strongly varying signals such as joint reaction forces is a challenging task. The question that often remains is if “characteristics” (such as local minima, local maxima or signal slopes) show a distinct pattern or if they appear randomly [23]. Dynamic time warping (dtw) has been established by Bender and Bergmann in order to compute typical signals (TS) which are aiming to provide the best representation of time series [23]. Parameters gathered during the dtw computations are also a measure of signal similarity. They represent different aspects of such the phase shift and the magnitudes, respectively. Healthy and able-bodied persons walk in a symmetrical way [24]. Following the concept of dynamic similarity, the time series of joint reaction force in healthy persons are also symmetrical [25]. Therefore an important outcome after THR is not only magnitude and orientation of hrf, but also symmetry of hrf as a measure to what extent gait pattern is pathological.

The purpose of the current study is to assess whether the artificial joint's hip reaction forces and patient's gait parameters can be improved by CAS FF THR by means of a combined workflow of experimental and computational methods relative to conventional THR. The specific target parameters were: (i) Are the hip reaction forces closer to a healthy, young normal in the CAS FF group? (ii) Are the hip reaction forces distributed more symmetrically in the CAS FF group? (iii) Is critical edge or rim-loading of the acetabular cup less likely to occur in the CAS FF group?

## 2. Patients and Methods

### 2.1. Patients

The study design, procedures, and informed consent were approved by our local medical ethics committee (number 10-121-0263). This single-center, patient- and observer-blinded randomized controlled trial was registered at the German Clinical Trials Register under the Main ID: (DRKS00000739).

Recruitment of participants, inclusion and exclusion criteria, and surgical procedures for this randomized controlled trial have been published prior to the start of the study [11]. Eligible participants between the ages of 50 and 75 with an American Society of Anesthesiologists (ASA [26]) score ≤3 were recruited from patients admitted for primary uncemented unilateral (minimal or no osteoarthritis in the opposite hip) THA due to primary or secondary osteoarthritis. Exclusion criteria were age <50 and >75 years, ASA score >3, coxarthrosis secondary to hip dysplasia, posttraumatic hip deformities, and prior hip surgery. Informed consent was acquired by one of four clinical investigators. THA in all patients was performed in the lateral decubitus position using a minimally invasive single-incision anterolateral approach. 160 THAs were performed by four orthopaedic surgeons from the Regensburg University Medical Center. Each surgeon had experience with more than 200 fluoroscopy and 200 navigation-controlled THAs. Press-fit acetabular components, uncemented hydroxyapatite-coated stems (Pinnacle cup, Corail stem, DePuy, Warsaw, IN), standard (nondysplastic and nonoffset) polyethylene liners and metal heads with a diameter of 32 mm were used in all patients.

**Figure 1:**
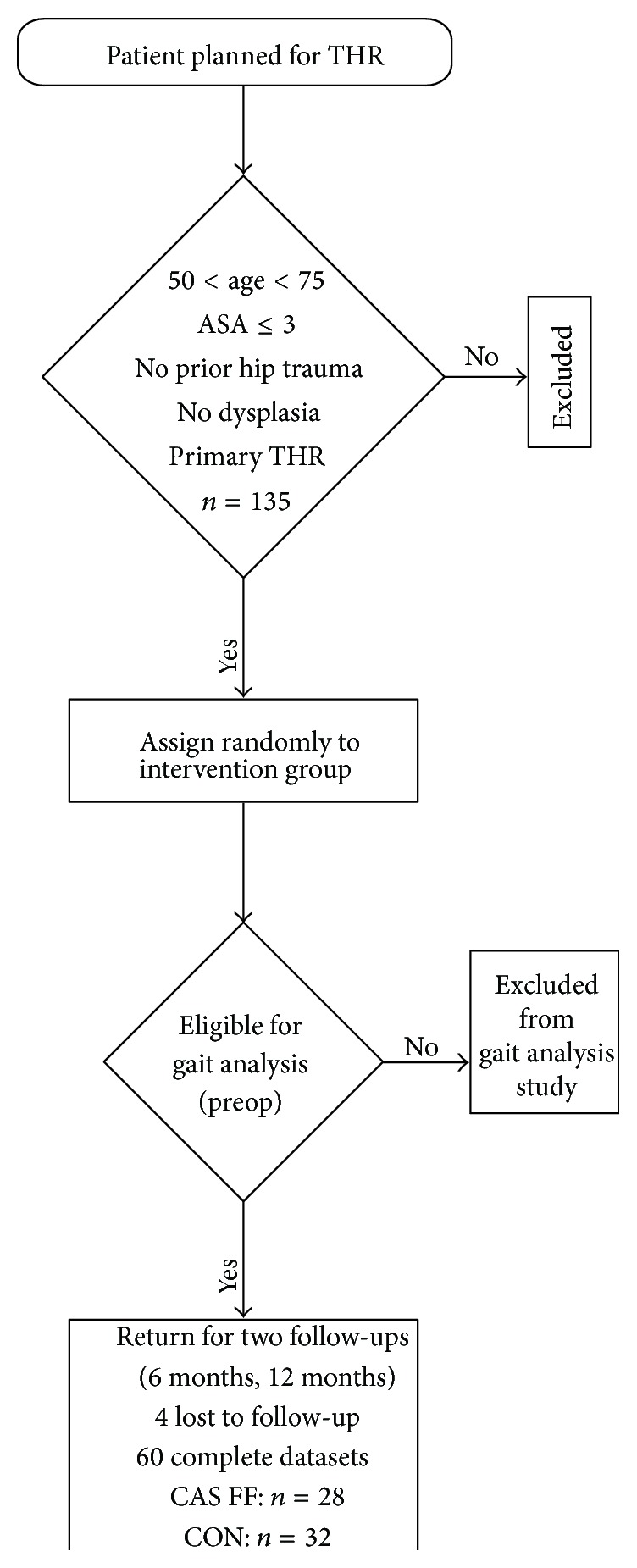
Flow chart of patient acquisition with inclusion criteria according to Renkawitz et al. [11].

Patients were randomly allocated to receive either Femur First CAS THR or conventional THR; see Figure 1. Patient characteristics according to allocation are presented in Table 1. The random allocation sequence was computer-generated in a permuted block randomization designed by the associate statistician using certificated randomization software (Rancode 3.6 Professional, IDV, Gauting, Germany).

### 2.2. Methods

#### 2.2.1. Computer-Assisted Minimally Invasive Femur First THR (CAS FF)

In the CAS Femur First group, an imageless navigation system (BrainLAB Navigation System Prototype Hip 6.0 “Femur First”, Feldkirchen, Germany) with newly developed prototype software was used [13].

#### 2.2.2. Conventional Minimally Invasive THR (CON)

Acetabular components were placed “freehand” without the use of any alignment guides. The target acetabular component position for all patients was within the “safe zone” as defined by Lewinnek et al. 18 (40° ± 10° inclination and 15 ± 10°, anteversion) [27].

#### 2.2.3. Gait Analysis (GA)

Sixty patients performed a 3D motion-capture (mocap) gait analysis of the lower extremity (SimiMotion, Unterschleißheim, Germany) at three time points (preoperative (*t*0), 6 months postoperative (*t*1), and 12 months postoperative (*t*2)). Only patients that were able to conduct a valid gait experiment (strike one force plate with one foot) were included in the GA-study. A bony and anatomical landmark based marker-set consisting of 27 retroreflective markers was previously tested to record the patient-specific gait pattern by means of six digital video cameras with a video sample rate of 70 Hz [28]. The patients walked at self-selected speed on a 10 m walkway, while the ground reaction forces were recorded simultaneously using two force plates (Kistler, Winterthur, Schweiz; sample rate: 1000 Hz). In order to calculate joint position based on marker data, a static trial was conducted before the gait experiment started. Prior to recording, the patients were asked to walk on the walkway three to five times in order to acquaint themselves with the laboratory situation. One patient missed* t*1-gait analysis but returned for the* t*2-analysis.

#### 2.2.4. Musculoskeletal Modeling (MM)

**Figure 2:**
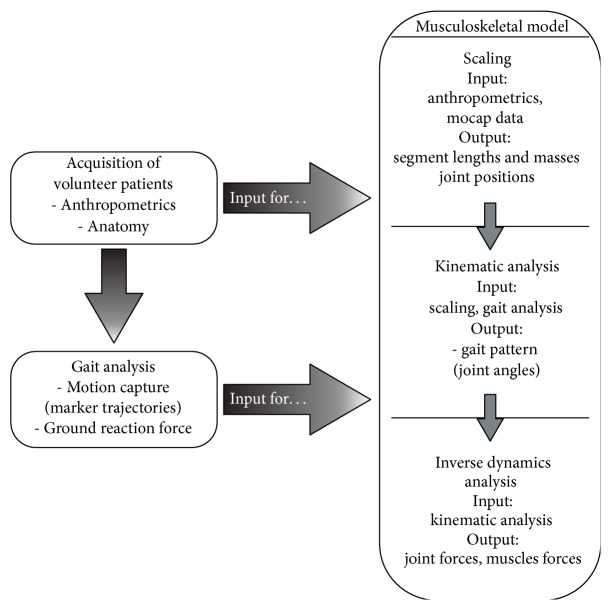
Study workflow combining experimental data with numerical simulations during gait.

The measured ground reaction forces and trajectories of the mocap markers retrieved during gait analysis were used as the input for the musculoskeletal model to compute the vectorial joint reaction forces during walking (Figure 2). Musculoskeletal analysis was conducted using a commercial software package (AnyBody Technology A/S, Aalborg, Denmark). A generic and previously validated model [29, 30] (AnyGait, AMMR1.6) was first scaled based on anthropometric measurements as an initial guess [31]. This was followed by a nonlinear scaling algorithm based on the maker data gathered during the static trial, further adapting the model to the patient specific anatomy [22]. The hip reaction forces were computed for one complete gait cycle with 150 computation steps for every model (Figure 2). The muscles were parameterized using the mechanical Hill-Type Muscle model, the tendons have been calibrated accordingly [32]. The time-dependent muscle activity is determined by a cubic optimization scheme and according to 1. Consider

(1) $$\begin{array}{c} G=\overset{}{\underset{i}{\sum }}{\left(\frac{\text{m}{\text{f}}_{i}}{N_{i}}\right)}^{3}, \end{array}$$

where *G* is the objective function to estimate muscle activation, mf is the muscle force vector (mf_*i*_ is the *i*th element) and *N* is the normalizing factor (muscle strength) [33]. *G* is to be minimized while the boundary conditions have to be satisfied (equilibrium fulfilled, muscles can only pull). The 179 MM in total were batch-processed with the aid of parallel computing, allowing eleven models to be computed at the same time using Matlab (Matlab Release 2013a, The MathWorks, Inc., Natick, Massachusetts, United States.).

#### 2.2.5. Method Verification

The measurement chain was evaluated with respect to different sources of variance (Table 2). Three healthy volunteer male subjects were invited (S1: 19 years, 79.4 kg, 1.73 m; S2: 25 years, 70.4 kg, 1.69 m; S3: 31 years, 73.4 kg, 1.82 m) to perform mocap gait analysis. The scope of the verification study was to evaluate the measurement chain and not to conduct a population study; hence such a narrow patient collective was acquired. Data was processed with the same workflow as for the patient study. To evaluate the measurement chain the standard error of mean (SEM) of the respective target parameter (Table 2) was computed according to 2 with *n* samples and a sample standard deviation *σ* [34]. Consider

(2) $$\begin{array}{c} \text{SEM}\left(\overset{-}{X}\right)=\frac{\sigma }{\sqrt{n}}. \end{array}$$

#### 2.2.6. Model Validation

The hrf retrieved from our patient cohort were compared to the publicly available hip 98 dataset (http://www.orthoload.com/) [17]. Hrf retrieved from healthy individuals were checked against literature data, which were obtained by using similar workflows [35].

#### 2.2.7. Dynamic Time Warping

While arithmetic means can only be formed at corresponding time-points *f*(*t*_*i*_), dynamic time warping is based on comparing every time-point *f*_1_(*t*_1,…,*k*_) to every time-point *f*_2_(*t*_1,…,*k*_). This is done by computing the dtw matrix according to Bender and Bergmann and according to 3. Consider

(3) df1i1,f2i2=α2f1i1f1−f2i2f22+1−α2f1′i1f1′−f2′i2f2′2.

**Figure 3:**
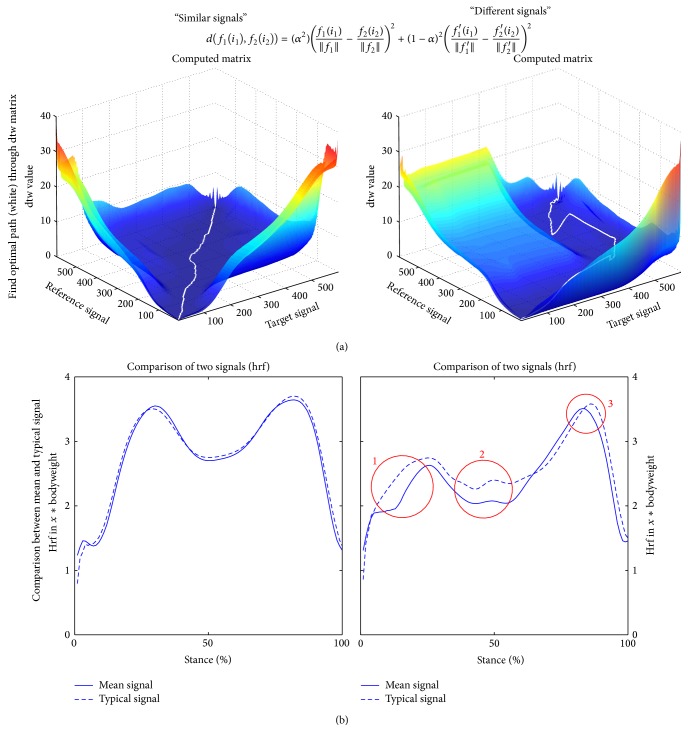
Using dtw for computing the TS from “similar” or “different” signals, respectively. (a) dtw matrix with optimized path (white) for similar (left) and varying (right) signals. (b) Comparison of mean signal (full line) and TS (dashed line) from the similar signals (left) and varying signals (right). For similar signals there is practically no difference between the mean signal and the TS. When comparing varying signals the TS yield different characteristics (red circles). Circle 1: no sharp cutoff, appears to be more harmonically, circle 2: peak is underestimated in mean signal, and circle 3: peak values between mean signal and TS are practically the same.

The two signals are then connected by minimizing the cumulated costs 4 along the “dtw path” (5, Figure 3) [23]. Consider

(4) $$\begin{array}{c} \text{CC}=\overset{N_{k}}{\underset{i_{k}=1}{\sum }}d\left(f_{1}\left(w\left(i_{k}\right)\right),f_{2}\left(w\left(i_{k}\right)\right)\right), \end{array}$$

(5) $$\begin{array}{c} w\left(i_{k}\right)=\left[\left(i_{1}\left(i_{k}\right)\right),\left(i_{2}\left(i_{k}\right)\right),i_{k}=1,2,\ldots ,N_{k}\right]. \end{array}$$

By minimizing the cumulated cost dtw takes the most “similar” values into account and permits the computation of a “typical signal” (TS) rather than comparing fixed time points *f*_1,2_(*t*_1,…,*k*_). While the difference between the mean signal and the TS is negligible when comparing “similar signals” (Figure 3), the strength of dtw is comparing varying signals, as it is often the case when comparing joint reaction forces during walking (Figure 3) [23]. By weighting the signals accordingly, it is also possible to compute the TS from more than two signals, as it was done for the comparison of the CAS FF and CON group.

The path length is a measure of phase shift between two signals (Figure 3). The cumulated cost along the path is a measure of magnitude similarity. Asymmetries are being computed between the operated and not-operated leg at* t*0,* t*1, and* t*2, as well as between the operated leg and a normative dataset at* t*0,* t*1, and* t*2.

#### 2.2.8. Postprocessing

**Figure 4:**
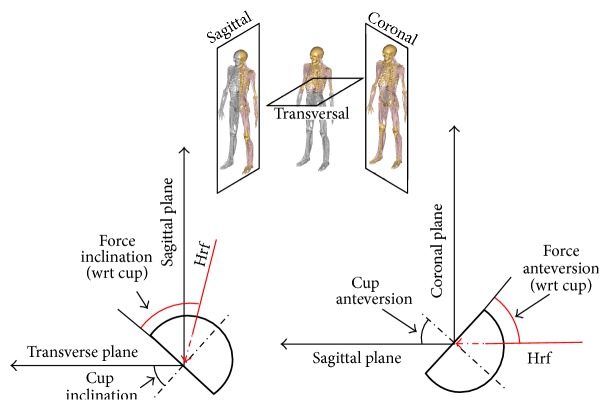
The definition of force orientation which is based upon the definition of acetabular orientation in the radiographic coordinate system according to Stansfield and Nicol [35].

Bagplots are used to visualize the distribution of bivariate statistical data [36]. The greater the area that is being enclosed, the wider the data is scattered and the more asymmetrical the patients walk in terms of hrf. Postprocessing was done using Matlab. Hrf orientations are quantified in the radiographic coordinate system according to Murray (Figure 4) [37].

#### 2.2.9. Statistics

ANOVA tests for unequal sample size (*n*_CASFF_ = 28, *n*_CON_ = 32) including group interactions were performed for all time-points (*t*0,* t*1,* t*2) divided by intervention groups on asymmetry parameters. Significance level was set at 5% (*α* = 0.05). Differences between intervention groups in terms of age, BMI, blood-loss, and operation time were tested using the student's *t*-test (*α* = 0.05) or with a chi-squared test (*α* = 0.05) for categorical data such as the ASA score.

## 3. Results

### 3.1. Patient—Characteristics

The groups showed no significant differences in terms of age, BMI, blood loss, and disease category (ASA-score). The operation time of the CAS FF group was significantly higher.

### 3.2. Method Verification

**Figure 5:**
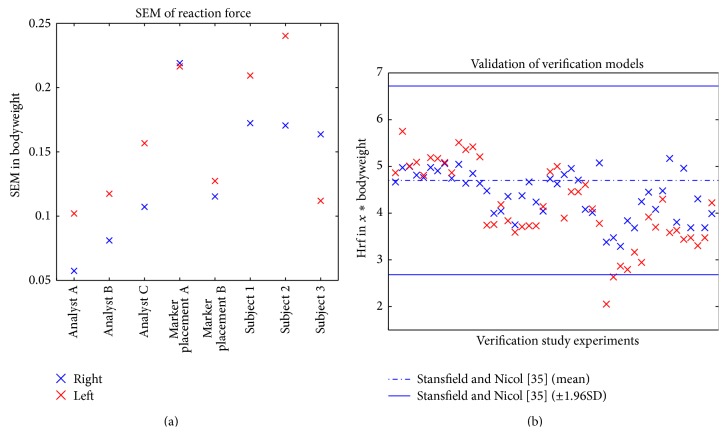
Results of the verification experiments. (a) SEM of hrf as computed by different verification studies. (b) Comparison of verification model with literature data [34] including 95% confidence interval (±1.96SD) of the literature data.

Figure 5 displays the verification study results. The mocap-analyst has a negligible influence on the target parameters. Marker Placement has the greatest influence on the target parameters. The repeatability study shows that results are indeed robust, but care must be taken when conducting experiments. A SEM of ±0.25 BW is an estimate of how accurate the hrf during walking can be computed. On the right hand side the maximum hrf of the normal subjects as computed with the aforementioned workflow are compared to literature (normal subjects, computed hrf) [35] (Figure 5). 97.8% of all models compute hrf that lie within the 95% (±1.96SD) confidence interval as published by Stansfield and Nicol [35]. We therefore considered the models valid for this study.

### 3.3. Model Validation

**Figure 6:**
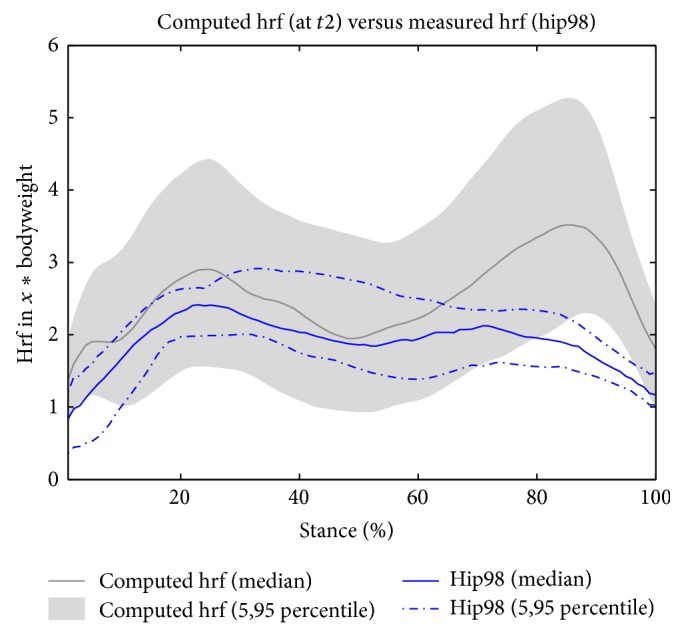
Validation study of patient models. The computed hrf are compared to the measured hrf (hip98-data). The hrf in multiples of bodyweight (*y*-axis) are displayed as a function of stance phase (0–100%, during walking). The gray area indicates the 5 and 95 percentile for (all) hrf (operated side) at* t*2, the dark gray line represents the median thereof. The blue lines represent either the hip98 median hrf (full line) or the 5 and 95 percentile (point-dashed line).

Figure 6 displays the comparison of computed hrf against measured hrf (hip98, http://www.orthoload.com/). Measured hrf were obtained from four subjects and between 11 and 31 months postop [17]; therefore the measured hrf are shown against the computed hrf at* t*2. The results show good agreement, especially at the first peak. The second peak seems to be overestimated by the computed hrf, but one should note that the measured hrf have been obtained by only four subjects, making valid statistical analysis challenging. The maximum hrf are up to twofold higher than measured ones; however such magnitudes have been reported for healthy subjects [35]. The models were considered valid for this study.

### 3.4. Typical Signal (TS) of Hrf

**Figure 7:**
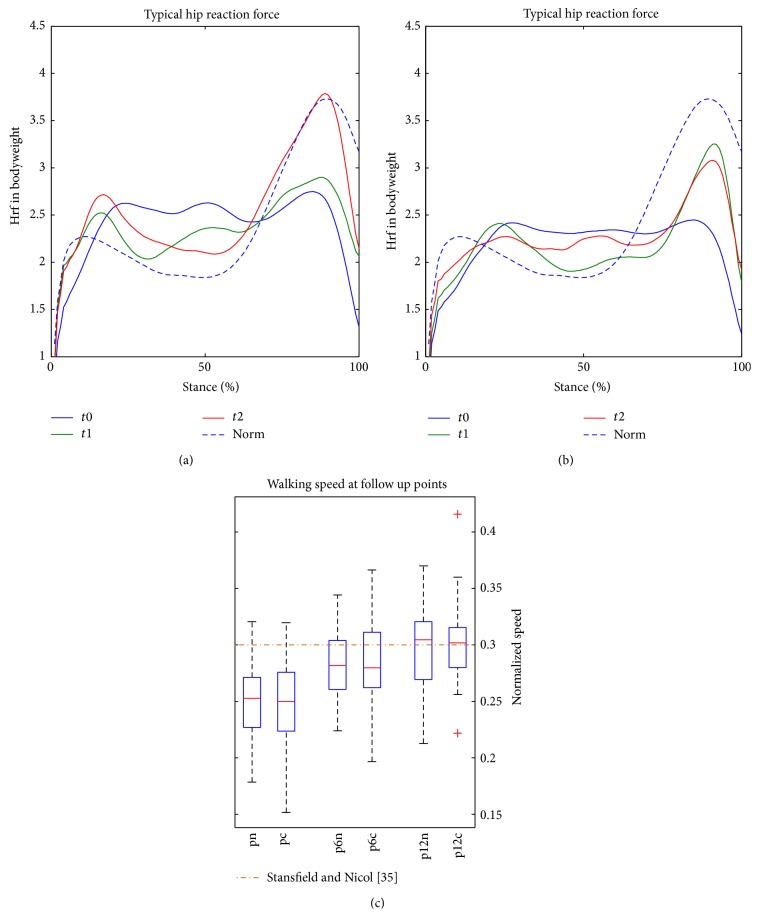
The TS of the different THR-groups. (a) The hrf of the CAS FF group at the different follow-up points can be seen (blue:* t*0, green:* t*1, red:* t*2). The *x*-axis denotes the stance-phase in percent; the *y*-axis shows the hrf in multiples of bodyweight. The dashed line is the TS of the healthy group as gathered during the method verification experiments. (b) Results for the CON group (blue:* t*0, green:* t*1, red:* t*2). (c) Dimensionless walking speed computed according to Hof [38] at different follow-up points is displayed, as well as literature data for THR patients [35] (point-dashed, dark red line).

The TS as computed by dtw including the normalized walking speed according to Hof are shown in Figure 7 [38]. The dimensionless walking speed increases significantly over all follow-up points, there are no significant differences between the two groups. While the hrf at* t*0 are in the same magnitude and similar shape for both groups, the hrf are increasing over the follow-up period. There are notable differences between the hrf at* t*1 for both groups, the hrf in the CON group are greater when compared to the CAS FF group (0.4 BW), bearing the SEM of ±0.25 BW in mind. At* t*2 the hrf of the CAS FF group are further increasing until becoming more similar to the healthy group in terms of magnitude and shape. At the second hrf peak of the CAS FF group there is practically no difference to data retrieved from young, healthy adults. The hrf of the CON group do not further increase.

### 3.5. Symmetries of Hrf

**Figure 8:**
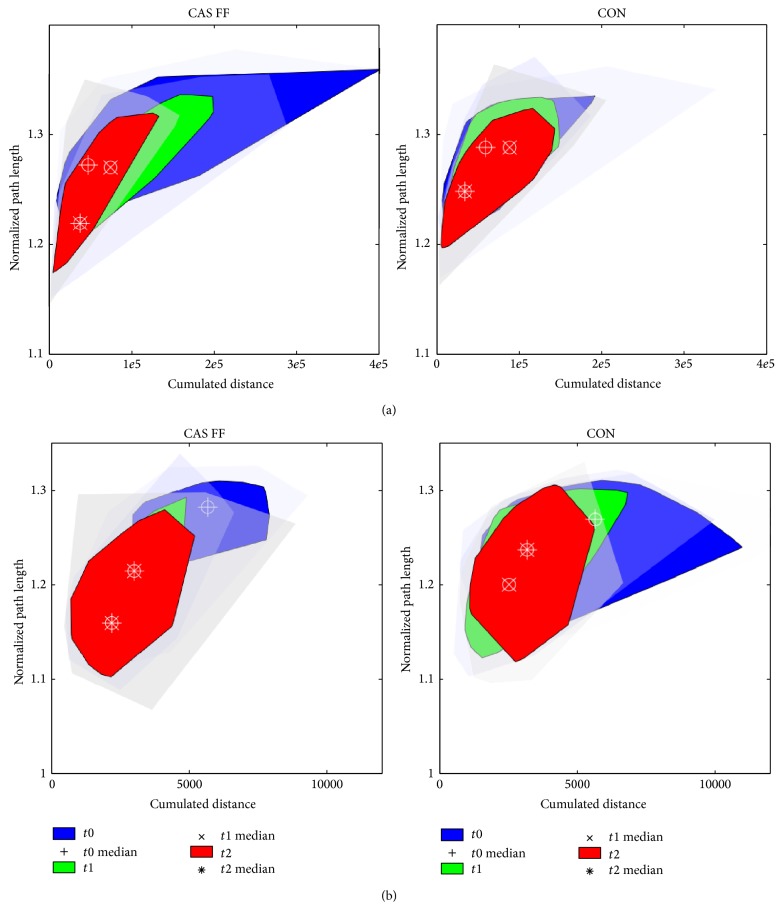
Bagplots of the deviation of joint reaction force time series as computed by dtw. On the left row the CAS FF group is displayed whereas on the right side the CON group is shown. On all *x*-axes one can see the cumulated distance as computed by 4 (magnitude similarity), whereas on all *y*-axes the normalized path length as computed by 5 (phase shift similarity) is displayed. (a) Comparison of hrf time series for operated versus not operated side at the follow-up points. (b) Comparison of hrf time series for operated versus normative data at the follow-up points.

Figure 8 displays the time series similarities of joint reaction forces as computed by dtw by means of bagplots [36].  Figure 8(a) shows the comparison of operated leg versus not-operated leg. Asymmetry measures are the greatest in the CAS FF group at* t*0; thus, those patients were walking preoperative more asymmetrical than patients in the CON group, but this was not significant. During the follow-up period both groups improve significantly in phase shift similarity as well as in magnitude similarity, thus patients are walking less asymmetrical at* t*2.  Figure 8(b) displays the joint-reaction force time series of the operated leg compared against the normative data. Preoperative values of the CAS FF group are not as scattered as for the CON group, but the difference was not significant. Phase shift and magnitude symmetry increases in both groups significantly, larger improvements can be found for the CAS FF group. In particular, phase shift similarity at* t*2 increased in the CAS FF group more than in the CON group which is also supported by the hrf-TS (Figure 7).

### 3.6. Orientation of Hrf at Peak Loads

**Figure 9:**
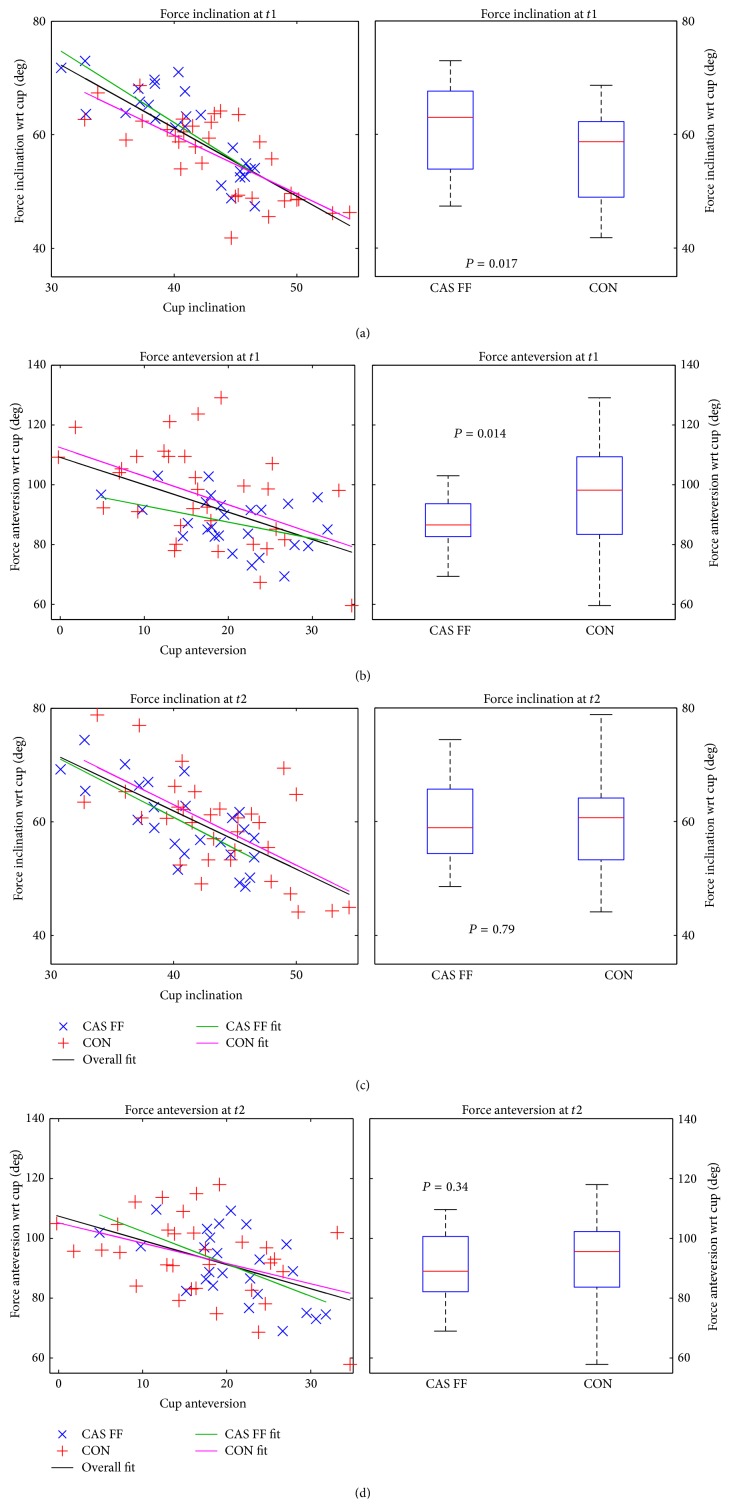
The hrf orientation at peak loads of the CAS FF and the CON group was compared. Left: hrf orientation as a function of cup orientation, including linear regression lines (black: overall regression; green: regression line for CAS FF group; magenta: regression line for CON group). Blue cross: CAS FF, red plus: CON. Right: boxplot of the hrf orientation at peak loads for the CAS FF and the CON group. (a) Force inclination at* t*1, (b) force anteversion at* t*1, (c) force inclination at* t*2, and (d) force anteversion at* t*2.

Figure 9 shows the force inclination and force anteversion at* t*1 and* t*2 respectively. The force inclination with respect to cup correlates significantly with the cup inclination in a linear fashion (*f*(*x*) = *a* · *x* + *b*) as does the anteversion at both follow-up points (Table 3). The coefficient of determination (*r* ^2^) is greatest at* t*1 for inclination of the CAS FF group (69% variance explained by linear model—Table 2). Roughly 25% variance is explained by the linear model for the anteversion angle. The variance explained for inclination decreases to 55% at* t*2 as does the correlation coefficient (−0.83→−0.74). At all follow-up points we performed a significance test (student's *t*-test, alpha = 5%) between the CAS FF and the CON group for the force angles. We found significant differences for both angles at* t*1, which vanished at* t*2. Patient that underwent CAS FF surgery showed force-angles closer to optimum (force angle = 90°—force attacks at center of hemisphere).  Table 4 lists the coefficients of the linear fit, showing good agreement for inclination angles. Deriving a linear regression line for the anteversion is also possible, but not with the same quality as for inclination (Table 3).

### 3.7. Orientation of Hrf at Peak Loads with respect to Combined Anteversion

**Figure 10:**
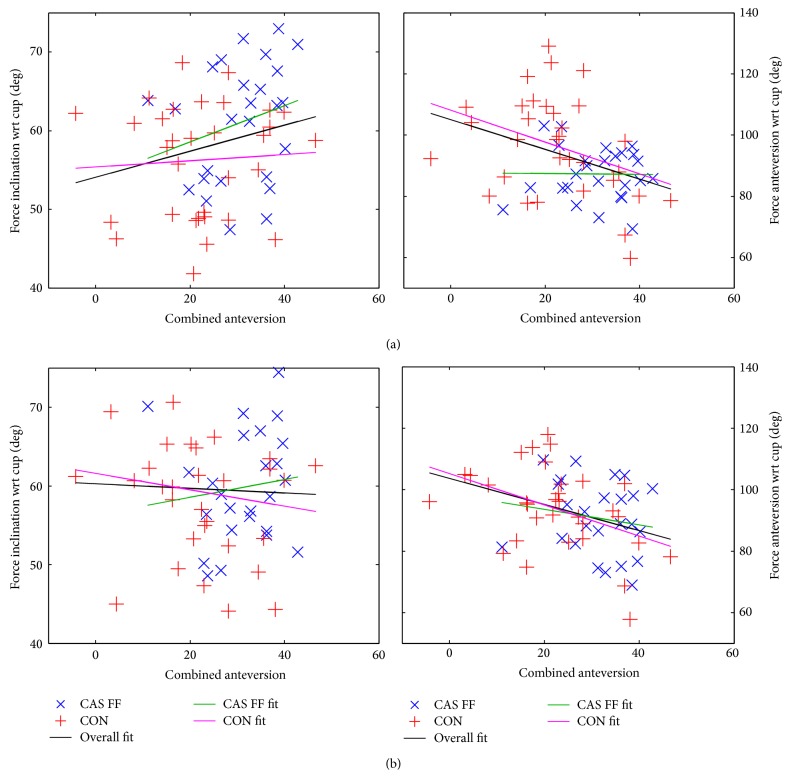
The hrf orientation at peak loads of the CAS FF and the CON group versus the combined anteversion. Left: hrf inclination as a function of combined anteversion, including linear regression lines (black: overall regression; green: regression line for CAS FF group; magenta: regression line for CON group). Blue cross: CAS FF, red plus: CON. Right: hrf inclination as a function of combined anteversion, including linear regression lines (a) force orientation at* t*1 and (b) force orientation at* t*2.

Figure 10 shows the force orientation (force inclination and force anteversion) as a function of the combined anteversion. There is no relationship between the force orientations of the CAS FF group (Table 5). The force anteversion of the CON group shows a significant but weak relationship to the combined anteversion (Table 5). The significant but weak relationship between the combined anteversion and the force anteversion for both groups is a result of the weak correlation for the CON group. Even if there is a relationship between combined anteversion and force orientation for the CON group, only a maximum of 13% of the variance of the data points can be explained, indicating once more influences of other unknown factors.

## 4. Discussion

The purpose of this study was to compare the hrf and their orientation between CAS FF and conventional THR.

The TS of the hrf shows improvement for the CAS FF group at the endpoint* t*2 when compared to normative data. Stansfield and Nicol report similar walking speed for THR patients postoperatively at comparable follow-up points [35]. Both groups performed approximately the same at* t*0, even if the asymmetries were greatest for the CAS FF group at* t*0. The fact that the hrf are decreasing between* t*1 and* t*2 in the CON group can be attenuated to measurement noise. Hrf, as an integral measure for muscle forces, are crucial for bone remodeling and bone in-growth [39, 40]. Therefore restoring the hrf to young healthy adult ones, as we observed it in our study for the CAS FF group, is the benchmark outcome for THR. Asymmetries of operated versus not-operated side in the CAS FF group decrease more than in the CON group, but the effect seems to be insignificant. It is important to also include the asymmetries of operated side versus normative data, since walking can also be symmetrical if both sides perform equally poor. The CAS FF THR group walks closer to a healthy normal, especially at* t*2 in terms of phase shift, meaning local maxima and minima are more likely to occur at the same time of a gait cycle as for a young healthy adult. This indicates a restored ability to walk [41] possibly resulting in a long-term benefit for the patients operated with CAS FF with functional optimization. Such long-term benefit remains to be proven which can only be achieved with additional follow-up points. At* t*1 the TS hrf of the CON group are closer to a healthy normal, but the force orientation for the CAS FF group is closer to optimum than in the CON group. Not only hrf magnitude is crucial for the implant survivorship, but also the orientation of the hrf [21]. Hrf that are closer to the edge of acetabular cup may result in edge or rim-loading, therefore increasing wear and compromising implant-survivorship [16]. While the hrf anteversion and inclination in the CAS FF group appears to be more favorable, the hrf orientation in the CON group is still noncritical, when taking into account that the inlay of the cup gets thinner on the edge (pinnacle/duraloc) due to manufacturing reasons [42]. Measurements taken from Effenberger and Hrsg [43] show that there are indeed cups where force orientation such as presented would result in rim-loading, but especially wear computations are necessary to identify critical regions in the cup [44] to back up this hypothesis. The significant difference in hrf orientation with respect to cup between the CAS FF and CON groups vanishes at* t*2, indicating that over the follow-up period the orientation as found in the CON group adjusts to similar hrf orientations found in the CAS FF group becoming closer to optimum. Our data suggests that the CAS FF procedure with functional optimization is especially beneficial at an early stage and has the potential to decrease the propensity for rim-loading and therefore dislocation and impingement within the first weeks after surgery. We also investigated a possible linear relationship between cup angle and force orientation and found a significant correlation between the force inclination and cup inclination at* t*1 with a high coefficient of correlation (−0.75). The other angles also revealed a nonzero relationship but the explained variance decreases over follow-up period (Table 3). This indicates that with a greater cup inclination and/or anteversion the angle between the resultant hip reaction force and the rim of the cup decreases. Therefore the resultant hip reaction force gets closer to the rim of the cup. This relationship becomes indeed valuable, when thinking of applying these results to either preoperational planning or real-time biomechanical feedback during surgery, which only CAS systems could provide. The fact that the correlation coefficient decreases over the follow-up period shows that other influences may play an important role (implant shaft, gait pattern) and that the relationship is multi-factorial. The weak or nonexistent relationship between the combined anteversion and force orientation supports this assumption. The force orientation cannot only be solely explained with implant orientation. This shows that for the definition of an implant safe-zone based on biomechanical evaluations the patient-specific anatomy and integral motion pattern is of vital importance. Based on such biomechanical evaluations a patient-specific optimal implant safe-zone may exist, which remains to be proven.

To the authors knowledge this is the first study that includes musculoskeletal models of gait in a prospective randomized controlled trial studying the computer-assisted femur first technique in relation to conventional THR. Other studies have been conducted to either study early outcome of standard CAS after THR by means of gait analysis [45] or to study the influence of surgical approach on gait parameters [46, 47]. The results of the gait analysis of these studies are mostly concise with our findings. There are no differences in temporospatial parameters such as walking speed or kinematic parameters. One study also compared muscle activation profiles of patients that underwent either MIS or conventional THR during walking which also did not reveal any significant differences [46]. MM have also been employed to study the outcome of THR but only on an individual basis or with a small, not randomized study population [16, 21, 48]. They all used the same validated musculoskeletal model and investigated similar parameters (edge-loading, total hrf). No evidence of occurring edge-loading in the studied population was found, which agrees with our findings.

### 4.1. Strengths

We investigated a rather large patient cohort, speaking for this kind of study (combining gait analysis with MM), which was also patient and observer blinded. To our knowledge this is the first study that has used this specific novel navigation algorithm in clinical practice. To the best of our knowledge this is also the first study ever with a patient- and observer-blinded, prospective randomized controlled study design on navigation in THR that has been published in the literature. The validated MM were highly patient-specific and we are confident that the models reflect the in vivo loads as accurate as possible using such a workflow. Comparing strongly varying signals by means of dtw has the advantage to not only focus on particular time points of signal time series. It rather compares all time-points to all time-points. This makes subjective and observer based decisions obsolete. To our knowledge and based on Bender and Bergmann [23] the TS as computed by dtw is the best representation of typical patterns as they occur in time series.

### 4.2. Limitations

Since the MM are purely mechanical models, psychological effect are cancelled out. By also analyzing clinical outcome scores (such as the Harris Hip Score (HHS) or the hip osteoarthritis outcome score (HOOS)) we tried to counter such effects; however the scores revealed no differences at* t*1 or* t*2, respectively. The movement of the upper body has not been quantified using motion capture. The measurement volume of the mocap is too small to effectively capture the movement of the body segments above the pelvis. The integral movement of the upper body has been approximated by mechanically balance the center of mass above the pelvis. This does not however reflect the movement, it is rather an approximation. We also did not include the patients' physical activity (PA) particularly. Evaluating the individual PA is very challenging, since the methods to evaluate PA are biased or can be deceived easily [49]. Gait performance in the laboratory may reflect the level of PA in and that is how we included this effect. Patients with higher levels of PA may recover faster than others. Therefore the results may be biased due to PA level of every particular patient. This is an effect that can only be countered by evaluating large patient cohorts. Even though the biomechanics are crucial for the functioning of one of the largest and weight-bearing joints in the human body, it is not completely sure that improved biomechanics also leads to an improved clinical outcome. Current research however shows that improved biomechanics leads to an improved outcome for the patient [50, 51]. Also biomechanical parameters have been found to be clinically relevant [52].

### 4.3. Clinical Relevance

We suggest practical application for our work such as operational planning based on biomechanical parameters (preop gait pattern, orientation of cup versus hrf orientation) by deriving simple laws and algorithms from the data. The results can also be used for real-time biomechanical feedback during CAS which is a scope for further research. A new safe-zone for implant component position and orientation is also thinkable, which relies on an accurate statistical model. This would draw the focus of implant positioning rather on analytical laws then experience. Future research will also include detailed finite element models based on patient specific medical imaging data and patient-specific muscle forces and boundary conditions. Such modeling will give more insight into implant bone contact stresses and wear in the hip joint, both are important for the prediction of implant survivorship.

## 5. Conclusion

The computer-assisted THR method following the concept of femur first/combined anteversion does lead to an improved outcome in contrast to conventional THR six month after surgery. In Particular, force orientation is close to optimum for the novel CAS Femur First technique at an early stage. A trend for decreased asymmetries of the gait pattern of the CAS FF group compared to the CON group indicates a restored walking ability and therefore a possible long-term benefit for the patients; however this hypothesis can only be proven by repeating the experiments at additional follow-up points.

## Figures and Tables

**Table 1:** Patient characteristics by intervention group.

Group	Sample size (male/female)	Age in years: mean (SD)	Age range: min/max	BMI ([kg/m^2^]): mean (SD)	BMI range: min/max	ASA mode (frequency)	Blood loss [g/dL]: mean (SD)	OP-time [minutes]: mean (SD)
CAS FF	28 (10/18)	60 (7)	50/74	26.73 (4.26)	19.45/35.22	2 (21)	−3.0 (1.0)	71 (15)
CON	32 (19/13)	62 (8)	50/74	27.58 (3.08)	21.64/33.68	2 (12)	−3.3 (1.2)	63 (13)

**Table 2:** Different sources of variance of the measurement chain and the studies in order to determine the standard error of mean.

Research question	Source of variance	Study	Target parameter
Is the result obtained dependent on the mocap analyst?	Mocap analyst	One healthy subject (S1), 1 gait analysis, evaluated 10 times by 3 different examiners: A (experienced), B (experienced), and C (not experienced)	Standard error of mean (SEM) of hrf-HRF_SEM_

How big is the influence of marker-placement on the results obtained?	Mocap—marker placement	One healthy subject (S1), 10 gait analyses, application of marker set in alternating manner by 2 analysts: A (experienced) and B (experienced)	HRF_SEM_

Is the method robust enough to produce repeatable results?	Measurement chain	Three healthy subjects (S1, S2, and S3), 10 gait analyses, evaluated by 1 experienced analyst (A)	HRF_SEM_

**Table 3:** Measure for the relationship between cup orientation and force orientation wrt cup at *t*1 and *t*2.

	Overall	CAS FF	CON
*t*1			
Inclination			
Pearson correlation coefficient (*R*)	−0.79	−0.83	−0.74
Significance of correlation (*P*)	9.0*e* − 14	1.3*e* − 07	1.3*e* − 06
Anteversion			
Pearson correlation coefficient (*R*)	−0.49	−0.41	−0.48
Significance of correlation (*P*)	8.6*e* − 05	0.039	5.8*e* − 03
*t*2			
Inclination			
Pearson correlation coefficient (*R*)	−0.66	−0.74	−0.64
Significance of correlation (*P*)	1.7*e* − 08	2.1*e* − 05	7.2*e* − 05
Anteversion			
Pearson correlation coefficient (*R*)	−0.48	−0.58	−0.41
Significance of correlation (*P*)	0.0001	0.002	0.019

**Table 4:** Fit parameters for the linear model *f*(*x*) = *a* · *x* + *b* for force orientation wrt cup orientation at *t*1 and *t*2  *x*[cup_degree_].

	*a* (force_degree_/cup_degree_)	*b* (degree)
*t*1		
Inclination		
Overall	−1.2	109.3
CAS FF	−1.4	116.8
CON	−1.0	101.1
Anteversion		
Overall	−0.9	109.2
CAS FF	−0.6	98.4
CON	−1.0	112.4
*t*2		
Inclination		
Overall	−1.0	102.9
CAS FF	−1.1	105.2
CON	−1.1	105.6
Anteversion		
Overall	−0.8	107.3
CAS FF	−1.1	113.0
CON	−0.7	105.2

**Table 5:** Measure for the relationship between combined anteversion and force orientation wrt cup at *t*1 and *t*2.

	Overall	CAS FF	CON
*t*1			
Force inclination			
Pearson correlation coefficient (*R*)	0.23	0.24	0.06
Significance of correlation (*P*)	0.08	0.23	0.74
Force anteversion			
Pearson correlation coefficient (*R*)	−0.37	−0.01	−0.36
Significance of correlation (*P*)	0.00	0.95	0.04
*t*2			
Force inclination			
Pearson correlation coefficient (*R*)	0.03	0.12	0.00
Significance of correlation (*P*)	0.84	0.56	0.99
Force anteversion			
Pearson correlation coefficient (*R*)	−0.31	−0.16	−0.35
Significance of correlation (*P*)	0.02	0.44	0.047
